# Effects of Tylosin, a Direct-Fed Microbial and Feedlot Pen Environment on Phenotypic Resistance among Enterococci Isolated from Beef Cattle Feces

**DOI:** 10.3390/antibiotics11010106

**Published:** 2022-01-14

**Authors:** Sarah A. Murray, Ashlyn C. Holbert, Keri N. Norman, Sara D. Lawhon, Jason E. Sawyer, Harvey M. Scott

**Affiliations:** 1 Department of Veterinary Pathobiology, Texas A&M University, College Station, TX 77843, USA; smurray@cvm.tamu.edu (S.A.M.); slawhon@cvm.tamu.edu (S.D.L.); 2 Department of Veterinary Integrative Biosciences, Texas A&M University, College Station, TX 77843, USA; aholbert@cvm.tamu.edu (A.C.H.); knorman@cvm.tamu.edu (K.N.N.); 3 Department of Animal Science, Texas A&M University, College Station, TX 77843, USA; j-sawyer@tamu.edu

**Keywords:** probiotic, *Enterococcus faecium*, antimicrobial resistance, environmental change

## Abstract

In two sequential replicates (*n* = 90 and *n* = 96 feedlot finisher cattle, respectively) we measured the impact of an *Enterococcus faecium*-based probiotic (DFM) and an altered feedlot pen environment on antimicrobial resistance among fecal enterococci in cattle fed (or, not fed) the macrolide tylosin. Diluted fecal samples were spiral-plated on plain and antibiotic-supplemented m-*Enterococcus* agar. In the first replicate, tylosin significantly (*p* < 0.05) increased the relative quantity of erythromycin-resistant enterococci. This effect was diminished in cattle fed the DFM in conjunction with tylosin, indicating a macrolide susceptible probiotic may help mitigate resistance. A similar observed effect was not statistically significant (*p* > 0.05) in the second replicate. Isolates were speciated and resistance phenotypes were obtained for *E. faecium* and *E. hirae*. Susceptible strains of bacteria fed as DFM may prove useful for mitigating the selective effects of antibiotic use; however, the longer-term sustainability of such an approach remains unclear.

## 1. Introduction

While the use of antibiotics for growth promotion was banned in the United States in 2016, they are still widely used for the prevention, control, and treatment of disease. Antibiotics, including tylosin and chlortetracycline, are used in feed for the prevention and control of liver abscesses in cattle. Grain-rich diets are widely accepted as the main cause of ruminal acidosis, rumenitis, and subsequent liver abscess formation [1,2]. Although it remains unknown as to any one specific causative agent and the exact pathogenesis for liver abscesses, *Fusobacterium necrophorum* is commonly isolated from abscessed livers, as is *Trueperella pyogenes*. It has been suggested that these two organisms initiate a synergistic reaction in causing the formation of liver abscesses [3]. Recently, certain *Salmonella enterica* serotypes such as Lubbock have been isolated during anaerobic culture from a liver abscess [1,4].

The greatest economic impact of liver abscesses results from reduced animal performance and decreased carcass yield. Cattle with abscessed livers have reduced feed intake, reduced weight gain, decreased feed efficiency, and decreased carcass dressing percentage [5]. Feed intake and feed conversion are impacted by severe liver abscesses, reducing intake by 5% and gain-to-feed by 14% [5]. A study by Brown et al. [6] also reported that adhesions increased the loss in HCW (hot carcass weight) by 3 kg in one comparison, and by 8.7 kg in a second comparison. They also reported a reduction in marbling score, in addition to reductions in yield grade, fat depth, and percent of kidney-pelvic-heart (KPH) fat in cattle with severe liver abscesses versus cattle with normal livers [7]. Additionally, a meta-analysis on liver abscess risks of cattle receiving tylosin versus cattle not receiving tylosin in conventional feeding systems showed that the feeding of tylosin reduced the risk of liver abscesses from 30% to 8% [8].

Meanwhile, tylosin has been shown to select for macrolide resistance when used as a growth promoter in swine [9]. In cattle, tylosin has also been associated with increased resistance to macrolides among fecal enterococci [10,11]. A systematic review of tylosin use in cattle estimated that when fed at approved dosages for typical durations, tylosin tended to increase the proportion of macrolide-resistant enterococci in cattle, thus suggesting a potential zoonotic risk to human beef consumers [12]. Additionally, in recent surveillance of all enterococci across the One Health continuum, *Enterococcus hirae* was the most common species isolated from cattle, followed by *Enterococcus villorum* and then *Enterococcus faecium* [13]. Furthermore, resistance to tetracycline and macrolides appeared to be abundant among the majority of enterococcal species, which the author posited as likely being due to the common use of antibiotics in both human and veterinary medicine [13].

This is of importance because *Enterococcus faecium*, though a common commensal bacterium, also is recognized as a leading opportunistic cause of nosocomial infections in intensive human health care settings [14]. In fact, *E. faecium* has been noted as being the second most prevalent nosocomial pathogen [15,16]. In the 2019 updated version of the Antimicrobial Resistance Threats Report by the U.S. Centers for Disease Control and Prevention, *E. faecium* was identified as the most common cause of central line-associated bloodstream infections [17]. While *E. faecium* is less likely to possess virulence factors than *E. faecalis*, it is more likely to carry a multi-drug-resistant geno- and phenotype [18].

Therefore, consideration of the possibility and impacts of shared mobile genetic elements among host-adapted strain of enterococci must be made. A problem arises when examining selected resistance to erythromycin in association with feeding tylosin, because it has been associated with the *erm* family of genes which confer resistance to a wide variety of macrolides, lincosamides, and streptogramin B. [10,11]. Additionally, there is the possibility of co-selection of resistance to tetracycline when feeding with tylosin, and vice versa [19]. Furthermore, macrolides such as erythromycin and azithromycin are key antibiotics in human health care, deemed to be in a category of the highest priority and of critical importance by the World Health Organization [20].

In response to these concerns, several studies have been performed exploring ways to limit tylosin use, or else to find non-antibiotic alternatives for liver abscess prevention. In 2015, Beukers et al. [21] suggested that antibiotic withdrawal prior to slaughter contributed to a reduction in the proportion of macrolide-resistant enterococci. Additionally, in 2018, Muller et al. [22] showed no difference in resistance among fecal enterococci from cattle fed with intermittent tylosin supplementation versus continuous treatment, thereby suggesting that environmental factors may be most important in carrying over resistance from one lot of cattle to the next, even more so than contemporaneous selection occurring during the actual feeding period.

In another direction, *Saccharomyces cerevisiae* fermentation products (SCFP) have been suggested for use to prevent liver abscesses, although there have been no statistically significant differences reported among treatment groups with respect to abscess prevalence or severity scores [23]. *Enterococcus faecium* is a unique bacterium, in that it can be deployed as a probiotic, especially due to its bile tolerance and its bacteriocins, which are antagonistic towards pathogenic organisms such as *Listeria monocytogenes* [24]. Furthermore, previous molecular work on these samples and the supplemented probiotic product has shown the DFM to be pan-susceptible, ST296. This sequence type was not present in fecal or manure pack samples prior to supplementation; however, it was found in fecal samples, and desiccated manure pack samples taken 120 days after the initial trial ended. The increase in prevalence of ST296 occurred in tandem with a decrease in ST240, the dominant sequence type associated with resistance genes *tet*(M) and *erm*B [25]. Therefore, by studying both the independent and interactive combined effects of an *Enterococcus faecium*-based direct-fed microbial (probiotic), tylosin and pen environmental change on phenotypic resistance in enterococci, we report their effects on the following objectives: (1) log_10_ overall and resistant *Enterococcus* CFU, (2) trends in resistance to a wide array of antibiotics, and (3) the significance of phenotypic macrolide resistance of select isolates.

## 2. Materials and Methods

### 2.1. Experimental Design

**Figure 1:**
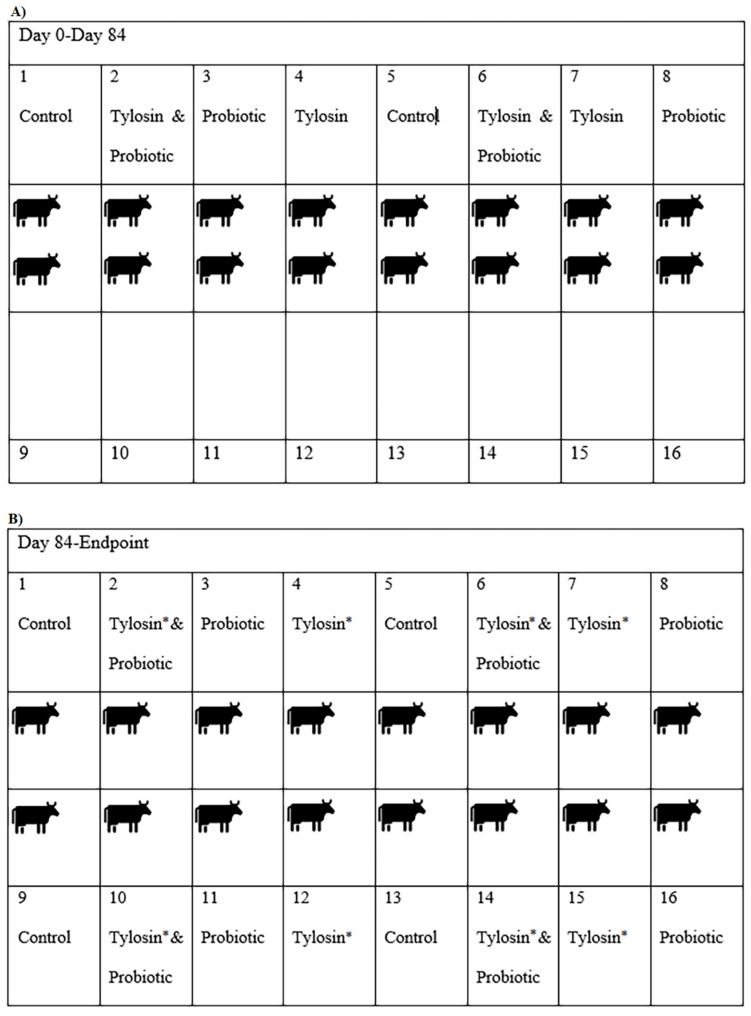
(**A**) Pen layout for first 12 weeks. Each cartoon figure represents six cattle. (**B**) Pen layout for last 4 weeks. Pens 1–8 were the ‘old’ pens, Pens 9–16 were the purpose-built ‘new’ pens. * Cattle were not fed tylosin from this point forward.

A randomized and controlled field trial consisting of two serial replicates in a 2 × 2 × 2 factorial design was conducted at the Texas A&M Agri-Life Research experimental feedlot in McGregor, Texas. Research was ethically and biomedically sanctioned, with approval of the Agriculture Animal Care and Use Committee and biosafety laboratory permits (AACUC AUP #2015-026A; IBC #2017-049 and #2017-021). The factors in the study were (1) tylosin, (2) DFM and (3) an environmental change to new pens. This resulted in a tylosin group, a DFM group, a combined tylosin/DFM group. Additionally, each treatment group interacted with the environmental change factor. This facility was unique, in that it previously had 8 cattle pens in use for many years; in addition, 8 new pens were purpose-built for this study in which no antibiotics had ever been used or fed, nor had they housed animals that had previously been treated with any antibiotic. Antibiotic-free and grass-fed beef cattle were allowed access to the new pens to prepare a manure pack during the 4 weeks prior to the trial; importantly, this was designed to ensure that a homogenous baseline of fecal bacteria was present in the newly constructed pens prior to the beginning of the feeding trial. The DFM used (Tri-Lution, Agri-King, Fulton, IL) contained 1.3 × 10^7^ CFU/g of *Enterococcus faecium* and *Saccharomyces cerevisiae*. The DFM was top-dressed into the feed for the proper treatment group. At the onset of the trial, steers were placed in the old pens and randomly assigned to a treatment group, with these treatments being: (1) tylosin (Tylan, Elanco, Greenfield, IN) included at 7.3 g/tonne), (2) DFM (824.5 g/tonne), (3) both tylosin and DFM, and (4) neither tylosin nor DFM (control). These four treatment groups were repeated in serial duplicates, so each treatment group had a replicate (Figure 1A). Four weeks prior to slaughter (hereafter referred to as the withdrawal timepoint), tylosin was removed from the respective trial ration while keeping the DFM feeding regimen for the appropriate treatment groups; meanwhile, half the steers in each of the four treatment groups were randomly assigned to a newly constructed adjacent pen purpose-built for this study (Figure 1B). For replicate 1, the trial ran for 112 days, and for replicate 2, the trial ran for 119 days. Hereafter the endpoint for the combined replicate data will be referred to as Day 119.

**Figure 2:**
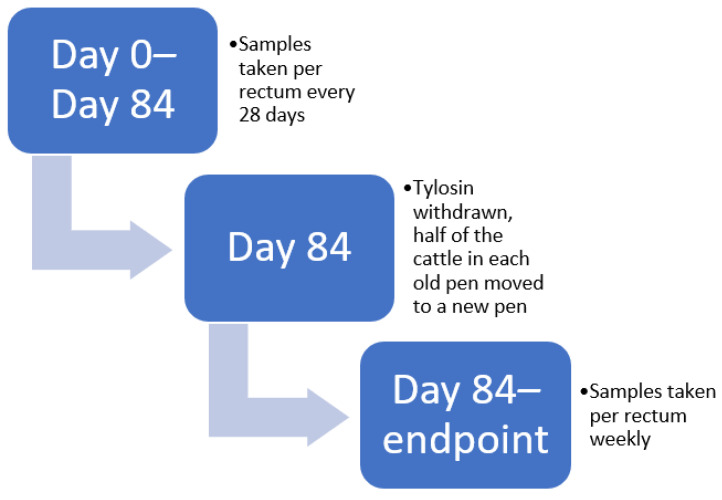
Timeline diagram of clinical stages of the study.

The first replicate consisted of 90 steers, while the second replicate consisted of 96 steers, all sourced from the same ranch birth cohort. Once every 28 days, in the morning, fecal samples were collected per rectum using new individual rectal palpation sleeves for 3 months (i.e., from Day 0 to Day 84) by the McGregor, Texas feedlot crew. Following Day 84, starting at the tylosin withdrawal time point, samples were taken weekly until slaughter (Figure 2). Samples were transported directly to the laboratory on ice immediately following the completion of collection. Samples were stored in the refrigerator until the next day, at which time they were processed. Sample processing consisted of aliquoting fecal samples into two 5 mL tubes; specifically, into one tube without glycerol and one tube with sterile 50% glycerol at a 1:1 ratio of glycerol to feces. Tubes were preserved at −80 °C until further use. This sample collection schedule, processing scheme, and storage was repeated for each of the two trial replicates.

### 2.2. Bacterial Enumeration, Isolation, and Speciation

Samples from Day 0 as the pretrial baseline measure, Day 84 as the antibiotic withdrawal time point (i.e., presumed maximum cumulative effect), and slaughter as the final time point were used for these analyses. Samples preserved with glycerol were thawed on ice and mixed thoroughly with phosphate buffered saline (PBS) (Gibco Life Technologies, Thermo Scientific Microbiology, Oakwood Village, OH, USA) using 4.5 milliliters of PBS to 0.5 g of feces to create a 1:10 dilution. An aliquot of 50 microliters of this dilution was spiral-plated onto plain m-*Enterococcus* agar (Difco, Becton Dickinson, Sparks Glencoe, MD), and onto m-*Enterococcus* agar supplemented with tetracycline and erythromycin at their Clinical and Laboratory Standards Institute (CLSI) human clinical breakpoints of 16 and 8 mg/L, respectively, using an EddyJet 2 Spiral Plater (Neutec Group Inc, Farmingdale, NY, USA). Plates then were incubated at 42 °C for 48 h.

Colony counts on each plate were performed using the Flash & Go^®^ System (Neutec Group Inc, Farmingdale, NY, USA). Two colonies presumptive for *Enterococcus faecium* (i.e., dark red to maroon with a cream halo) were selected from each of the plain and erythromycin-supplemented plates when possible. When presumptive *Enterococcus faecium* isolates were not available, presumptive *Enterococcus hirae* was selected instead. Colonies were quadrant-streaked for isolation onto tryptic soy agar (TSA) with 5% sheep blood agar (Remel^™^, Lenexa, KS, USA) and incubated at 37 °C for 24–48 h. A single colony from each TSA agar with 5% sheep blood plate was again isolated and streaked fresh onto TSA with 5% sheep blood agar and incubated at 37 °C for 24–48 h and then saved for further analysis.

Each saved isolate was subjected to MALDI-TOF for the confirmation of genus and species. Using a new sterilized wooden toothpick per isolate, a single isolate of presumptive *Enterococcus faecium* or *Enterococcus hirae* was spread onto two wells of a reusable 96-well target plate (Bruker Daltonik GmbH., Billerica, MA, USA). Once dry, one microliter of 70% formic acid was added to the first well of each sample spot pair of each *Enterococcus* spp. isolate, in addition to one empty spot to serve as a negative control. One microliter of the bacterial test standard (BTS) solution (Bruker Daltonik GmbH., Billerica, MA, USA) was applied to the first and second wells as a positive control. After drying all wells, one microliter of HCCA matrix solution (Bruker Daltonik GmbH., Billerica, MA, USA) was added to each well, including all the sample wells, BTS wells, formic acid negative control well, and an additional empty well as a secondary negative control. The target plate was then transferred to the MALDI-TOF Microflex LT/SH for reading, using MBT Compass v1.4 software (Bruker Daltonik GmbH., Billerica, MA, USA).

### 2.3. Phenotypic Susceptibility Testing

To obtain minimum inhibitory concentrations, microbroth dilution using the Sensititre^®^ (TREK, Thermo Scientific Microbiology, Oakwood Village, OH) platform was used. Isolates were freshly plated to TSA with 5% sheep blood agar and incubated at 37 °C for 18–24 h. Afterward, 11 mL of sterilized water was normalized to a 0.5 McFarland standard. Next, 10 μL of the culture suspension was transferred to 11 mL of sterile Mueller–Hinton broth. Subsequently, 50 μL of the broth culture was inoculated into each well of the NARMS Gram-positive CMV3AGPF plate for *Enterococcus* spp. using the Sensititre^®^ automated inoculation delivery system (TREK, Thermo Scientific Microbiology, Oakwood Village, OH, USA). The plate consisted of 16 antibiotics from 13 classes, including: chloramphenicol, ciprofloxacin, daptomycin, erythromycin, gentamicin, kanamycin, lincomycin, linezolid, nitrofurantoin, penicillin, quinupristin/dalfopristin, streptomycin, tetracycline, tigecycline, tylosin, and vancomycin (Table 1).

Plates were incubated at 37 °C for 24 h, with *Escherichia coli* ATCC 25922, *Escherichia coli* ATCC 35218, *Pseudomonas aeruginosa* ATCC 27853, *Staphylococcus aureus* ATCC 29213, and *Enterococcus faecalis* ATCC 29,212 serving as quality controls. Plates were read using a Sensititre OptiRead^™^ instrument (TREK, Thermo Scientific Microbiology, Oakwood Village, OH, USA). The results were interpreted as susceptible (S), intermediate (I), or resistant (R) in accordance with CLSI guidelines according to the M100 document [26], and NARMS breakpoints when CLSI breakpoints were not available, using SWIN software (TREK, Thermo Scientific Microbiology, Oakwood Village, OH, USA).

### 2.4. Statistical Analysis

The prevalence of enterococci was modeled using log_10_ transformed CFU per gram of feces bacterial count data as the dependent variable in the full factorial multilevel mixed effect linear regression model. The independent factors in the model used were: (1) tylosin (binary), (2) DFM (binary), and (3) sample day (integer). Trial replicate and original pen number were treated as nested random effects. No tylosin treatment, no DFM treatment, and day 0 were used as a baseline. A similar approach has previously been employed for log-transformed quantitative plate count data in a cattle experimental design [27]. Due to the low percentage of growth on erythromycin-supplemented agar plates, a Cragg hurdle regression model was used for the analysis of the dependent variable of log_10_ CFU per gram of feces for erythromycin-resistant enterococci, using the same independent factors as the multilevel mixed effect linear regression. The linear selection hurdle model was fit for the bounded dependent variable (growth on erythromycin-supplemented agar), combined with an outcome model for nonbounded values, using the same covariates. For statistical analysis of phenotypic resistance using Sensititre^™^, isolates interpreted as intermediate were reclassified as susceptible, yielding a binary variable (resistant/susceptible). Each antibiotic class was treated as a dependent variable in a full factorial multilevel mixed effect logistic regression model to determine significant increases or decreases in resistance. The independent factors used in the model were: (1) tylosin (binary), (2) DFM (binary), and (3) sample day (integer), with the trial replicate and original pen as nested random effects. No tylosin treatment, no DFM treatment, and day 0 were used as a baseline.

## 3. Results

### 3.1. Descriptive Statistics

Of the 558 fecal samples, 270 samples were collected from Trial Replicate 1 and 288 fecal samples were collected from Trial Replicate 2. From Replicate 1, 98.5% (*n* = 266) of the samples were quantifiable on plain m-*Enterococcus* agar, while 83.7% (*n* = 226) of the samples were quantifiable on tetracycline-supplemented m-*Enterococcus*, and 31.1% (*n* = 84) of the samples were quantifiable on erythromycin-supplemented agar. In Replicate 2, 99.3% (*n* = 286) of the samples were quantifiable on plain m-*Enterococcus* agar, while 90.3% (*n* = 260) of the samples were quantifiable on tetracycline-supplemented m-*Enterococcus*, and 49.0% (*n* = 141) of the samples were quantifiable on erythromycin-supplemented agar. The CFU per gram of feces was normalized using a log_10_ transformation. For statistical analyses, samples exhibiting no growth were recorded as zero (0). Samples which were not quantified exhibited no growth (no samples were coded as too numerous to count—TNTC).

### 3.2. Mixed Multivariable Regression Models

For the quantification of total enterococci, a multi-level linear regression was performed on the log_10_ CFU per gram of feces colony counts, in a 2 × 2 × 2 full factorial design, with the factors being tylosin, DFM and sample day, with the pen and replicate as random effects.

**Figure 3:**
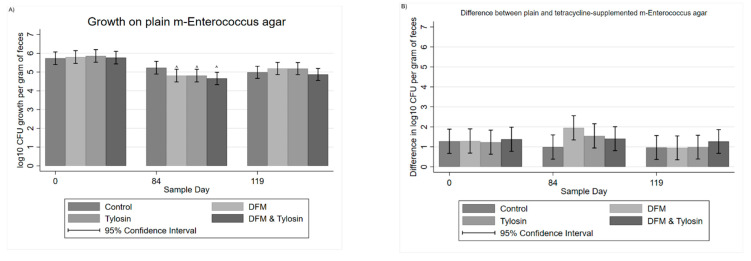

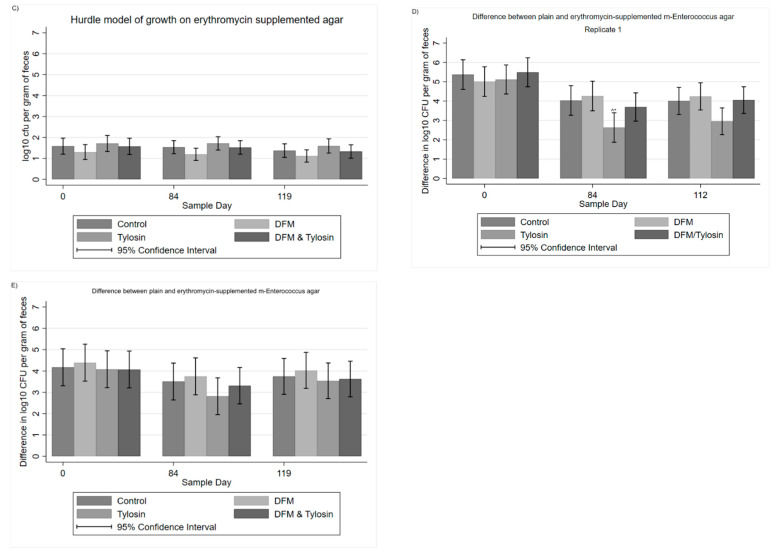
(**A**) Mixed-model marginal mean log_10_ CFU per gram of feces on plain m-*Enterococcus* agar, ^ significantly different from respective treatment groups on Day 0; (**B**) mixed model difference in log_10_ CFU between plain and tetracycline-supplemented m-*Enterococcus* agar; (**C**) Cragg hurdle model estimates of log_10_ CFU per gram of feces on erythromycin-supplemented m-*Enterococcus* agar; (**D**) mixed model estimated differences in log_10_ CFU between plain and erythromycin-supplemented m-*Enterococcus* agar from Replicate 1, ^ significantly different from respective treatment group on Day 0 * significantly different from other treatment groups on Day 84, and (**E**) mixed model estimated differences in log_10_ CFU between plain and erythromycin-supplemented m-*Enterococcus* agar from both replicates.

Period effects significantly impacted the log_10_ CFU per gram of feces from Day 0 to Day 84 among the DFM, tylosin, and combined DFM/tylosin groups. The DFM group, tylosin group, and combined DFM/tylosin group significantly (*p* < 0.05) decreased in log_10_ CFU per gram of feces from Day 0 to Day 84 (Figure 3A); however, the control tended to decrease, though was not significantly different from Day 0. By subtracting the log_10_ growth on tetracycline-supplemented agar from the corresponding growth on plain agar, the resulting difference is interpretable as follows: the size of the difference is inversely related to levels of antibiotic resistance, such that a decrease in the difference between plain and tetracycline-supplemented agar should be interpreted as an increase in resistance. The difference between plain and tetracycline-supplemented agar illustrated a decrease in the proportion of resistance for the DFM group (Figure 3B); however, this was not statistically significant (*p* > 0.05). For growth on erythromycin-supplemented agar, Cragg’s hurdle model was used to account for the high number of zero counts (Figure 3C). The DFM group tended to have slightly lower counts on erythromycin-supplemented agar, but was not significantly different from any other treatment group. Additionally, none of the treatment-specific temporal changes were statistically significant (*p* > 0.05).

Comparing the difference between plain and erythromycin-supplemented agar, in the first replicate alone, there was a significantly decreased difference (*p* < 0.05), and therefore increased resistance, in the tylosin group on Day 84 as compared to Day 0 (Figure 3D). Additionally, on Day 84, the tylosin group was significantly different (*p* < 0.05) from the DFM group. In contrast, the combined tylosin/DFM group was not significantly different from the tylosin group, the DFM group, or the control on Day 84. However, in replicate 1 on Day 112, 4 weeks after half the cattle were moved to new pens and the withdrawal of tylosin from feed, the tylosin-fed group still showed a significantly decreased difference between plain and erythromycin-supplemented agar when compared to Day 0. This was not significantly different from Day 84, and likewise was not significantly different (*p* > 0.05) compared to any of the other treatment groups on Day 112. When both replicates were combined, treating both the pen and replicate as random effects, there remained a tendency towards a decreased difference in CFU on plain and erythromycin plates in the tylosin group on Day 84 (Figure 3E). However, this was not statistically significant (*p* > 0.05). Additionally, when both replicates were combined, the tylosin group was not significantly different from the DFM group on Day 84.

### 3.3. Descriptive Statistics of Phenotypic Resistance

**Figure 4:**
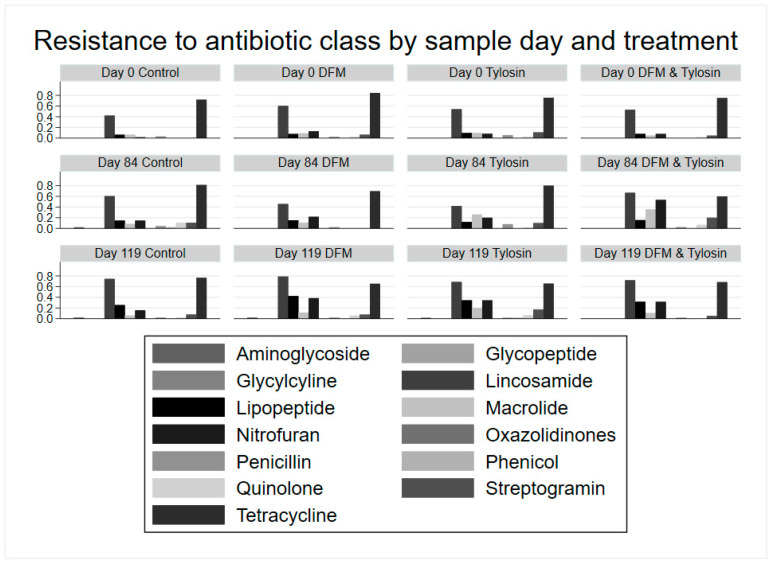
Resistance (proportion) of *Enterococcus* spp. isolates to each antibiotic class by sample day and treatment group.

Regarding the phenotypic resistance of *Enterococcus* spp. isolates, all 693 isolates of either *E. faecium* or *E. hirae* isolated from plain m-*Enterococcus* agar were susceptible to gentamicin, linezolid, tigecycline, and vancomycin (Figure 4). A majority of isolates, 73.3%, were resistant to tetracycline. Additionally, over 50% of the tetracycline-resistant isolates were right-censored with respect to minimum inhibitory concentration (MIC), and thus continued to grow at the highest concentration of 32 mg/L. Over half of the isolates, a total of 59.6%, were resistant to lincomycin. Only 11.5% of isolates were resistant to erythromycin. Less than 25% of the isolates were resistant to daptamycin or nitrofurantoin. Less than 10% of isolates were resistant to chloramphenicol, ciprofloxacin, kanamycin, penicillin, streptomycin, quinupristin/dalfopristin, or tylosin. Of the 8.7% of isolates specifically resistant to tylosin, 7.7% grew at the highest concentration of 32 mg/L, and were therefore right-censored with respect to MIC (Table S1).

Resistance of *Enterococcus* spp. isolates to each antibiotic class by sample day and treatment showed a trend towards increasing resistance to macrolides in the tylosin group and the combination tylosin/DFM group (Figure 4). Macrolide resistance decreased in both tylosin-fed groups (following withdrawal) from Day 84 to Day 119. Additionally, there was decreased resistance to tetracycline among isolates in the DFM group and combination tylosin/DFM group. However, the tylosin group increased in resistance from Day 0 to Day 84, and subsequently decreased after tylosin withdrawal. The increase in macrolide resistance, and the pattern of both increased and decreased resistance to tetracycline was later tested for statistical significance using multi-level mixed logistic regression.

### 3.4. Multi-Level Mixed Effects Logistic Regression Modeling of Antibiotic Resistance Phenotype

**Figure 5:**
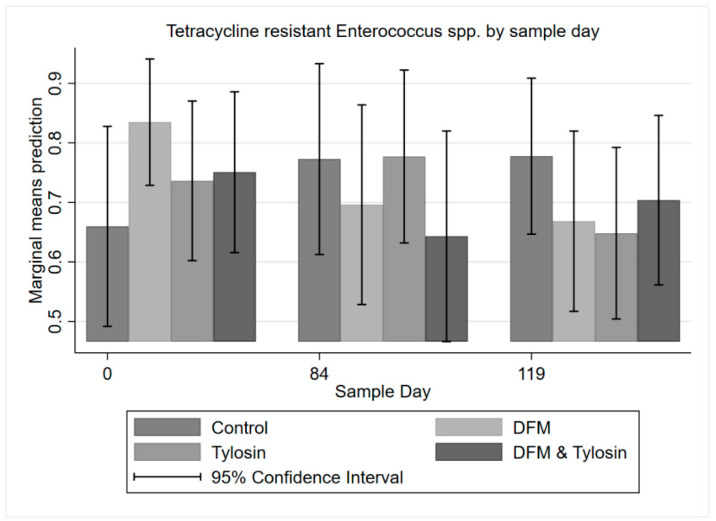
Marginal means with 95% confidence intervals of a 2 × 2 × 2 multi-level mixed logistic regression model, using factors of DFM, tylosin, and sample day on the binary outcome of tetracycline-resistant *Enterococcus* spp.

A multi-level mixed effects logistic regression on tetracycline-resistant enterococci with binary endpoints showed a trend towards decreased resistance to tetracycline from Day 0 to Day 84 for the DFM and combination tylosin/DFM groups; however, this was not statistically significant (*p* > 0.05). Additionally, there was decreased resistance to tetracycline in the tylosin group from Day 84 to Day 119 (following product withdrawal); however, these differences were also not significant (Figure 5).

**Figure 6:**
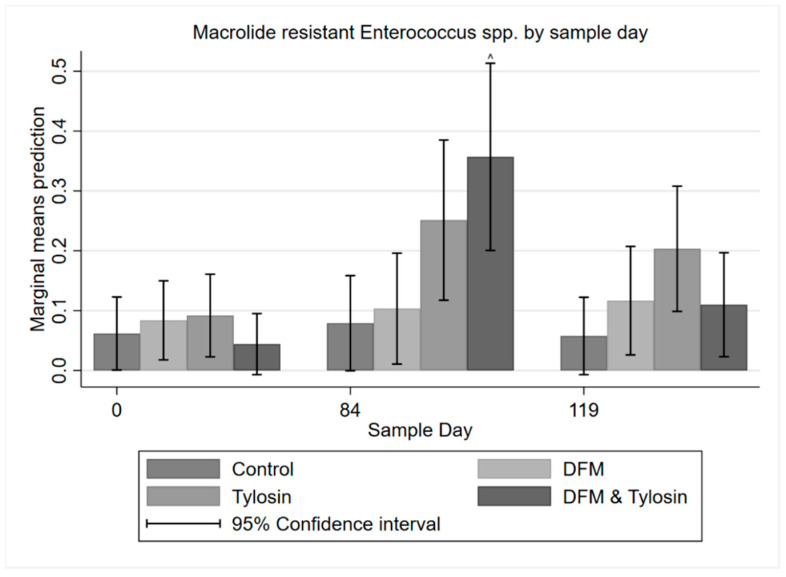
Marginal means with 95% confidence intervals of a 2 × 2 × 2 multi-level mixed logistic regression model, using factors of DFM, tylosin, and sample day on the binary outcome of macrolide resistant *Enterococcus* spp. ^ Significantly different from respective treatment group on Day 0 and Day 119.

A multi-level mixed effects logistic regression on erythromycin resistance among enterococci isolates with binary endpoints showed a trend of significantly (*p* < 0.05) higher resistance to macrolides in the combination DFM/tylosin group on Day 84 compared to Day 0. Additionally, after the withdrawal of tylosin, resistance to macrolides significantly decreased in the combination group from Day 84 to Day 119 (Figure 6). The tylosin group showed a similar increase from Day 0 to Day 84; however, the differences were not statistically significant (*p* > 0.05).

## 4. Discussion

### 4.1. Host Bacterial Response to Antibiotics and Direct-Fed Microbials

Overall, there was a significant difference (*p* < 0.05) in the quantification of enterococci between plain and erythromycin-supplemented agar in the first replicate for those pens receiving tylosin alone. This difference was no longer significant (*p* > 0.05) 4 weeks after the withdrawal of tylosin at the end of the trial, and this effect was not present in any of the other groups, namely the combined tylosin/DFM group. This suggests the DFM may have had an attenuating effect on macrolide resistance when fed in conjunction with tylosin. This supports previous molecular sequencing data from these samples, in which the prevalence of ST296, the macrolide-susceptible probiotic sequence type, increased among fecal samples over the trial period, concomitant with the decrease in prevalence of ST240, which was associated with resistance genes *erm*B and *tet*(M) [25]. Additionally, the isolates from tylosin/DFM group also had significantly more phenotypic resistance to macrolides on Day 84 when compared to Day 0, and less resistance was found at slaughter compared to Day 84. This trend of increased resistance after a period of time being fed tylosin, and then a decrease following its withdrawal, corresponds with previous results from Beukers et al. [21], in that the withdrawal of tylosin prior to slaughter contributes to a decrease in macrolide-resistant enterococci.

Remarkably, when the phylogeny of multidrug-resistant *E. faecium* was traced by Lebreton et al., it was found that while the emergence of the hospital-adapted lineage occurred in association with the early human use of antibiotics, the bacterial populations at that time consisted of a majority of animal-derived strains and was not associated with human commensals [28]. The same authors traced an earlier bifurcation of *E. faecium*, the divergence of human and animal strains (3000+ years ago), to a time corresponding with the emergence of domestic agriculture, including the keeping of livestock and specialized diets.

### 4.2. Role of the Pen and Ambient Environment

A systematic review by Cazer et al. [12] concerning the effects of tylosin on antimicrobial resistance in beef cattle found synthesized evidence that tylosin feeding increased the proportion of macrolide-resistant enterococci in the gastrointestinal tract. When tylosin is fed in conjunction with the ionophore monensin, enterococci have also been shown to exhibit increased resistance to macrolides [10]. However, it has been suggested that more than simple antibiotic use is at play, as a study by Jackson et al. [29] showed that while macrolide-resistant *Enterococcus* spp. were higher on a farm which used tylosin, they were still present on another farm which did not use tylosin. This implies that the environment must play an important role in sustaining resistance, and its magnitude therefore likely reflects the scale of historical use. As suggested previously by Muller et al., in a study in which cattle that were not fed tylosin did not have significantly fewer macrolide-resistant enterococci than cattle which were consistently fed tylosin [22], both continuously and intermittently fed groups (and a negative control group) showed a significant increase in erythromycin resistance from arrival in the feedlot until late in the feeding period that could not be attributed to concurrent feeding of tylosin. The results of Muller et al. [22] suggest that a history of environmental tylosin use significantly affects macrolide resistance among enterococci. With respect to the environmental impact on resistance in the results of this study, macrolide resistance among isolates in the DFM/tylosin group was significantly different on Day 84 at the height of tylosin treatment, when compared to the baseline Day 0 and the endpoint Day 119. This indicates that a withdrawal of tylosin, the continuation of a macrolide-susceptible probiotic, and an environmental change to new pens may affect macrolide resistance among enterococci isolates. Additionally, the significantly increased relative quantity of erythromycin-resistant enterococci in the tylosin group on Day 84 of replicate 1 compared to the combined tylosin/DFM group implies that the DFM has a mitigating effect on erythromycin resistance.

Others have asserted that tylosin minimally affects resistance in beef cattle, and suggested that resistance may be seasonal; however, it should be noted that in the month in which the tylosin-treated cattle exhibited a higher prevalence of macrolide-resistant enterococci, the corresponding pen also had a higher prevalence of macrolide resistant *Enterococcus* spp. [30]. It was of interest in our study that the DFM was associated with decreased tetracycline resistance from Day 0 to Day 84, which occurred in both quantification and phenotypic resistance among isolates, although this was not significant.

### 4.3. Assumptions and Future Potential

A study by Amachawadi et al. [31] pointed out the potential problems with using commercial probiotics, including the isolation of multidrug-resistant *E. faecium* from the products. Even though the probiotic used in this study was not multidrug-resistant, it could still have significant impacts on antimicrobial resistance, be subjected to selection pressures, or conjugate plasmids with resistant bacteria, as *Enterococcus faecium* is known to readily share plasmids with *Staphylococcus aureus* [32], as well as members of its own genus or species. Therefore, the significantly decreased difference (and therefore increased resistance) shown in replicate 1 in macrolide resistance in the DFM group as compared to the tylosin group alone could imply that the DFM may mitigate this resistance among both log_10_ CFU per gram of feces quantification and enterococcal isolates. Additionally, the lack of a significantly decreased difference in macrolide resistance from Day 0 to Day 112 in both the DFM group and the combined DFM/tylosin group could suggest that the combination of a macrolide-susceptible probiotic, the withdrawal of tylosin before slaughter, and the movement of cattle may be a viable future alternative to combat erythromycin resistance in beef cattle fed with tylosin.

## 5. Conclusions

In conclusion, tylosin and its subsequent withdrawal have a measurable effect on macrolide-resistant enterococci during the cattle feeding period and macrolide resistance among isolates, which agrees with previous studies. While the results in our first replicate favored the use of a probiotic to mitigate erythromycin resistance among fecal enterococci, the results from the second replicate were inconclusive. The starting levels (Day 0) of macrolide resistance can differ between trial replicates, and whenever cattle spend extended periods of time in any feedlot environment, the levels of resistance will rise uniformly across all groups, thus reducing the potential for differences to emerge between treatment groups. Thus, trial replicate 1 may have differed from trial replicate 2. Meanwhile, other factors, such as season or age of cattle, may also be at play. Therefore, further studies to evaluate the use of an *Enterococcus*-based probiotic while accounting for pre-existing environmental conditions should be performed.

## Figures and Tables

**Table 1:** Antibiotics ordered by class, concentration range (mg/L) and interpretive breakpoint (for resistance) for the NARMS (National Antimicrobial Resistance Monitoring System) Gram-positive plate (CMV3AGP), using CLSI (Clinical and Laboratory Standards Institute) criteria and NARMS interpretive human breakpoints when a CLSI equivalent was unavailable.

Antibiotic	Class	Range	Breakpoint
Gentamicin	Aminoglycoside	128–1024	≥500
Kanamycin	Aminoglycoside	128–1024	≥1024
Streptomycin	Aminoglycoside	512–2048	>1000
Vancomycin	Glycopeptide	0.25–32	≥32
Tigecycline	Glycylcycline	0.015–0.5	≥0.5
Lincomycin	Lincosamide	1–8	≥8
Daptomycin	Lipopeptide	0.25–16	≥8
Erythromycin	Macrolide	0.25–8	≥8
Tylosin	Macrolide	0.25–32	≥32
Nitrofurantoin	Nitrofuran	2–64	≥128
Linezolid	Oxazolidinone	0.5–8	≥8
Penicillin	Penicillin	0.25–16	≥16
Chloramphenicol	Phenicol	2–32	≥32
Ciprofloxacin	Quinolone	0.12–4	≥4
Quinupristin/dalfopristin	Streptogramin	0.5–32	≥4
Tetracycline	Tetracycline	1–32	≥16

## Data Availability

The datasets used and/or analyzed are available from the corresponding author on reasonable request.

## Supplementary Materials

The following supporting information can be downloaded at: https://www.mdpi.com/article/10.3390/antibiotics11010106/s1. Table S1: Percentage of *Enterococcus* spp. isolates that were resistant and their distribution across minimum inhibitory concentrations (MIC) for each antibiotic. Black vertical lines indicate the human CLSI (or, NARMS) interpretive breakpoint, grey boxes indicate areas above and below the highest and lowest limits of the assay antibiotic concentrations, respectively. Isolates which exceeded growth at the highest antibiotic concentration were placed in the next MIC column (shown in the grey area).

## Institutional Animal Care and Use Committee Statement

The study was approved by the Agriculture Animal Care and Use Committee of Texas A&M University (Animal Use Protocol #2015-026A).
