# Supplementary material for: Effects of Tylosin, a Direct-Fed Microbial and Feedlot Pen Environment on Phenotypic Resistance among Enterococci Isolated from Beef Cattle Feces
Source: Antibiotics (Basel). 2022 Jan 14;11(1):106. doi: 10.3390/antibiotics11010106 (PMC8772914; doi:10.3390/antibiotics11010106)
Supplement: Supplementary file 1 [file antibiotics-11-00106-s001.zip › antibiotics-1500574-supplementary.pdf]

antibiotics

MDPI

	# Resistant (of 693 tested)	% Resistant	95% Confidence Interval		<0.015	0.015	0.03	0.06	0.125	0.25	0.5	1	2	4	8	16	32	64	128	256	512	1024	2048	>2048
Chloramphenicol	2	0.3	0.03	1.0									1.73	65.22	32.32	0.435	0.145	0.145						
Ciprofloxacin	22	3.2	2	4.8					0.58	7.94	47.04	30.45	10.82	3.03	0.145									
Daptamycin	124	17.9	15.1	21.0						0.87	0.43	2.45	13.85	64.5	16.88	0.575	0.435							
Erythromycin	80	11.5	9.26	14.2						48.92	24.1	2.16	5.19	8.18	4.04	7.5								
Gentamicin	0	0.0	0	0.53*															100					
Kanamycin	3	0.4	0.09	1.3															83.26	14.43	1.88	0.435		
Lincomycin	413	59.6	55.8	63.3								35.96	2.45	2.02	11.43	48.2								
Linezolid	0	0.0	0	0.53*							0.87	3.75	76.48	18.9										
Nitrofurantoin	141	20.4	17.4	23.5									1.3	0.29	0.435	18.9	28.72	30.01	20.35					
Penicillin	20	2.9	1.77	4.4						7.79	4.18	16.88	40.12	25.69	2.45	2.89								
Streptomycin	1	0.1	0	0.8																	99.86	0.145		
Quinupristin/Dalfopristin	57	8.2	6.29	10.5							39.25	5.05	47.47	7.94	0.145	0.145								
Tetracycline	508	73.3	69.8	76.6								22.37	1.88	0.87	1.59	3.17	18.04	52.09						
Tigecycline	0	0.0	0	0.53*		0.58	17.75	26.7	53.1	1.88														
Tylosin	60	8.7	6.67	11.0						0.435	0.72	8.51	61.33	13.28	5.77	1.3	1.01	7.65						
Vancomycin	0	0.0	0	0.53*						0.87	52.38	44.73	0.72	1.3										
*97.5% One-sided CI																								

Supplemental Table S1. Percentage of *Enterococcus* spp. isolates that were resistant and their distribution across minimum inhibitory concentrations (MIC) for each antibiotic. Black vertical lines indicate the human CLSI (or, NARMS) interpretive breakpoint, grey boxes indicate areas above and below the highest and lowest limits of the assay antibiotic concentrations, respectively. Isolates which exceeded growth at the highest antibiotic concentration were placed in the next MIC column (shown in the grey area).
